# Cardiovascular morbidity and mortality among persons diagnosed with tuberculosis: A systematic review and meta-analysis

**DOI:** 10.1371/journal.pone.0235821

**Published:** 2020-07-10

**Authors:** Christopher Andrew Basham, Sarah J. Smith, Kamila Romanowski, James C. Johnston

**Affiliations:** 1 Provincial TB Services, British Columbia Centre for Disease Control, Vancouver, Canada; 2 School of Population and Public Health, University of British Columbia, Vancouver, Canada; 3 Max Rady College of Medicine, Rady Faculty of Health Sciences, University of Manitoba, Winnipeg, Canada; 4 Department of Medicine, University of British Columbia, Vancouver, Canada; Rush University, UNITED STATES

## Abstract

**Introduction:**

The emerging epidemiological evidence of increased cardiovascular disease (CVD) risk among persons diagnosed with tuberculosis (TB) has not been systematically reviewed to date. Our aim was to review the existing epidemiological evidence for elevated risk of CVD morbidity and mortality among persons diagnosed with TB compared to controls.

**Materials and methods:**

EMBASE, MEDLINE, and Cochrane databases were searched (inception to January 2020) for terms related to “tuberculosis” and “cardiovascular diseases”. Inclusion criteria: trial, cohort, or case-control study design; patient population included persons diagnosed with TB infection or disease; relative risk (RR) estimate and confidence interval reported for CVD morbidity or mortality compared to suitable controls. Exclusion criteria: no TB or CVD outcome definition; duplicate study; non-English abstract; non-human participants. Two reviewers screened studies, applied ROBINS-I tool to assess risk of bias, and extracted data independently. Random effects meta-analysis estimated a pooled RR of CVD morbidity and mortality for persons diagnosed with TB compared to controls.

**Results:**

6,042 articles were identified, 244 full texts were reviewed, and 16 were included, meta-analyzing subsets of 8 studies’ RR estimates. We estimated a pooled RR of 1.51 (95% CI: 1.16–1.97) for major adverse cardiac events among those diagnosed with TB compared to non-TB controls (p = 0.0024). A ‘serious’ pooled risk of bias was found across studies with between-study heterogeneity (I2 = 75.3%).

**Conclusions:**

TB appears to be a marker for increased CVD risk; however, the literature is limited and is accompanied by serious risk of confounding bias and evidence of publication bias. Further retrospective and prospective studies are needed. Pending this evidence, best practice may be to consider persons diagnosed with TB at higher risk of CVD as a precautionary measure.

## Introduction

Tuberculosis (TB) is the world’s leading cause of death from an infectious disease with the highest incidence in low- and middle-income countries [1]. In 2018, 10 million persons were estimated to develop active TB with approximately 1.2 million deaths attributable to active TB among persons without known human immunodeficiency virus (HIV) [2]. The absolute number of deaths attributable to active TB has declined 27% (from 1.8 million) between 2000 and 2018, with an estimated 42% drop in the TB mortality rate [2]. With declining mortality and increasing survival beyond treatment, there is a growing need to consider the long-term health of persons surviving TB treatment [3–7]. If the *End TB Strategy’s* target of a 95% reduction in mortality from TB by 2035 is met [8], the importance of considering the long-term health of persons surviving TB treatment will rise dramatically.

Cardiovascular disease (CVD) is a leading cause of death worldwide with epidemic increases in rates among low- and middle-income countries [9]. Links between infectious diseases and CVD have been drawn in recent years [10]. A recent review of literature on pneumonia and myocardial infarction showed significantly increased short- and long-term risk of myocardial infarction in those with pneumonia compared to those without [11].

Given the chronic nature of TB, these links raise questions about the contribution of TB to CVD and its role as a potential risk factor (or marker) for CVD beyond traditional risk factors such as smoking, diet, and physical activity [10,12]. A recent narrative review described several plausible biological mechanisms for TB in CVD processes, including both active and latent TB [12], while a series of analyses from Taiwan investigated a range of CVD outcomes associated with TB, such as ischaemic stroke and acute coronary syndrome [13,14]. Acute myocardial infarction risk has also been linked to active and latent TB and other infectious diseases [11,15–17]. A systematic review of TB and hypertension did not find a significant association [18].

As survival among persons diagnosed with TB improves, post-TB health is becoming a priority for TB researchers, programs, and care providers [7]. While HIV programs have adopted guidelines for noncommunicable disease screening and care [19–21], similar guidance for patients diagnosed with TB is lacking. We therefore systematically reviewed the published literature on TB and CVD as a logical step towards evidence-based guidance.

Our objective was to critically appraise the epidemiological evidence for an association between TB and CVD. We sought to evaluate our hypothesis of elevated CVD among persons diagnosed with active or latent TB compared to the general population or suitable controls through a pooled relative risk (RR) estimate. In this study, CVD included death from, or diagnosis of, unstable angina, atherosclerosis, ischemic heart disease, coronary heart disease, myocardial infarction, ischemic stroke, hemorrhagic stroke, heart failure, cerebrovascular event, or peripheral arterial disease.

## Materials and methods

This systematic review and meta-analysis was designed and reported using the Preferred Reporting Items for Systematic Reviews and Meta-Analyses, as well as the Meta-Analysis of Observational Studies in Epidemiology checklist [22–24]. The study was prospectively registered [25]. We worked with a university librarian specializing in health research to develop our search strategy.

### Data sources and searches

Both MeSH and text words were employed in the search strategy. MeSH search terms included “cardiovascular diseases” and “tuberculosis”, including all subheadings for each, and various terms for trial, cohort, or case-control study designs (S1–S3 Tables). We also searched for specific cardiovascular diseases and variants of “tuberculosis” as text words based on our protocol [25]. The databases EMBASE®/Ovid®, MEDLINE®/PubMed® were searched for studies published between 1946 and January 17, 2020 (S1 and S2 Tables). The Cochrane Database of Systematic Reviews and CENTRAL Registry of Clinical Trials were searched using similar terms from inception to January 10, 2020 and December 2019, respectively (S3 Table). The *International Journal of Tuberculosis and Lung Disease* was manually reviewed from June 1, 2013 to December 1, 2019 for relevant studies. Reference lists of included studies were manually searched for additional studies.

### Study selection

We included published studies that: used trial, cohort or case-control study design; had a clinical or microbiological definition of TB; reported outcomes from a patient population that included persons diagnosed with TB; and reported a risk estimate of one or more types of CVD morbidity or mortality in persons diagnosed with TB compared to suitable control subjects with an estimate of precision. A study was excluded if it: did not provide a definition of TB for the population studied; did not provide a definition of the outcome(s) used in the study; was a duplicate study; did not have an abstract published in English; or did not involve human participants.

After duplicate articles were removed, two independent reviewers (CAB and SJS) screened titles and abstracts for relevance. The same two independent reviewers then reviewed the full text of all remaining studies, applying inclusion and exclusion criteria independently, with any disagreements resolved by a third reviewer (JCJ).

### Data extraction and quality assessment

Data required to describe the studies and conduct a meta-analysis were extracted and coded by two independent reviewers (CAB and SJS) using a common template. The extractions were reconciled through discussion and a third reviewer (JCJ) when necessary. From each study, we extracted first author’s surname, country or setting of study, year published, study objective, source of the study sample, time period for enrollment, type of RR measure reported, RR estimate and 95% confidence interval, number of persons diagnosed with TB, type of TB among the exposure group, follow-up time, the CVD morbidity and mortality outcome, whether pre-existing CVD was removed from analytic sample (or otherwise analyzed incident CVD), the proportion of study population with HIV, and adjustment variables for the RR estimate used in the meta-analysis.

Two reviewers (CAB and SJS) independently assessed included studies for risk of bias (RoB) within studies using the Risk of Bias In Non-randomized Studies of Interventions (ROBINS-I) tool, adapted to the observational studies included in our review and incorporating our protocol-anticipated concerns regarding confounding [25,26]. After RoB assessment was complete, reviewers arrived at an overall RoB judgment for each study by consensus. To provide a review-level RoB assessment, we calculated the mode of the overall RoB judgments across studies.

### Data synthesis and analysis

A subset of reviewed studies with sufficient data appropriate to answer the review question and from non-overlapping populations were considered for quantitative synthesis. To synthesize the summary measures across studies, we implemented an inverse-variance weighted meta-analytic approach to estimate a pooled RR and 95% confidence interval using the empirical Bayes estimator from a random effects model [27–29]. Between-study heterogeneity was quantified by the *I2* statistic and meaningfully presented in prediction intervals [30]. Forest plots were created displaying meta-analytic results.

We conducted both a per-protocol meta-analysis as well as a *post hoc* meta-analysis. The per-protocol meta-analysis adhered to the prospectively registered data analysis plan, and all sensitivity analyses flowed from this meta-analysis. A *post hoc* meta-analysis was conducted to provide a more interpretable pooled RR, restricted to active tuberculosis and major adverse cardiovascular events (MACE) including: cardiovascular mortality, acute myocardial infarction, unstable angina, and nonfatal stroke. However, because of the programmatic and clinical value of treating TB as a binary division of latent and active TB, we considered the *post hoc* meta-analysis as our main meta-analysis and base our final interpretations on this meta-analysis.

To assess the robustness of the findings from our per-protocol meta-analysis, we conducted three sensitivity analyses: first, we removed estimates from studies that required a medical condition other than TB for inclusion (i.e., HIV infection [31,32], and non-chest surgery [33]); second, we excluded estimates that were not adjusted for risk factors beyond age and sex; and third, we removed one estimate that was of extra-pulmonary TB. Two sub-group analyses were conducted, one analyzing only CVD mortality, and one analyzing only CVD events (acute myocardial infarction, ischaemic stroke, or cerebrovascular event).

Publication bias was assessed through construction of Galbraith (radial) and funnel plots [27,34]. We used linear regression (Egger’s) test and rank correlation test for asymmetry with α = 0.05 to judge significance of publication bias. All data analyses were conducted in *R* version 3.4.4.

## Results

**Fig 1:**
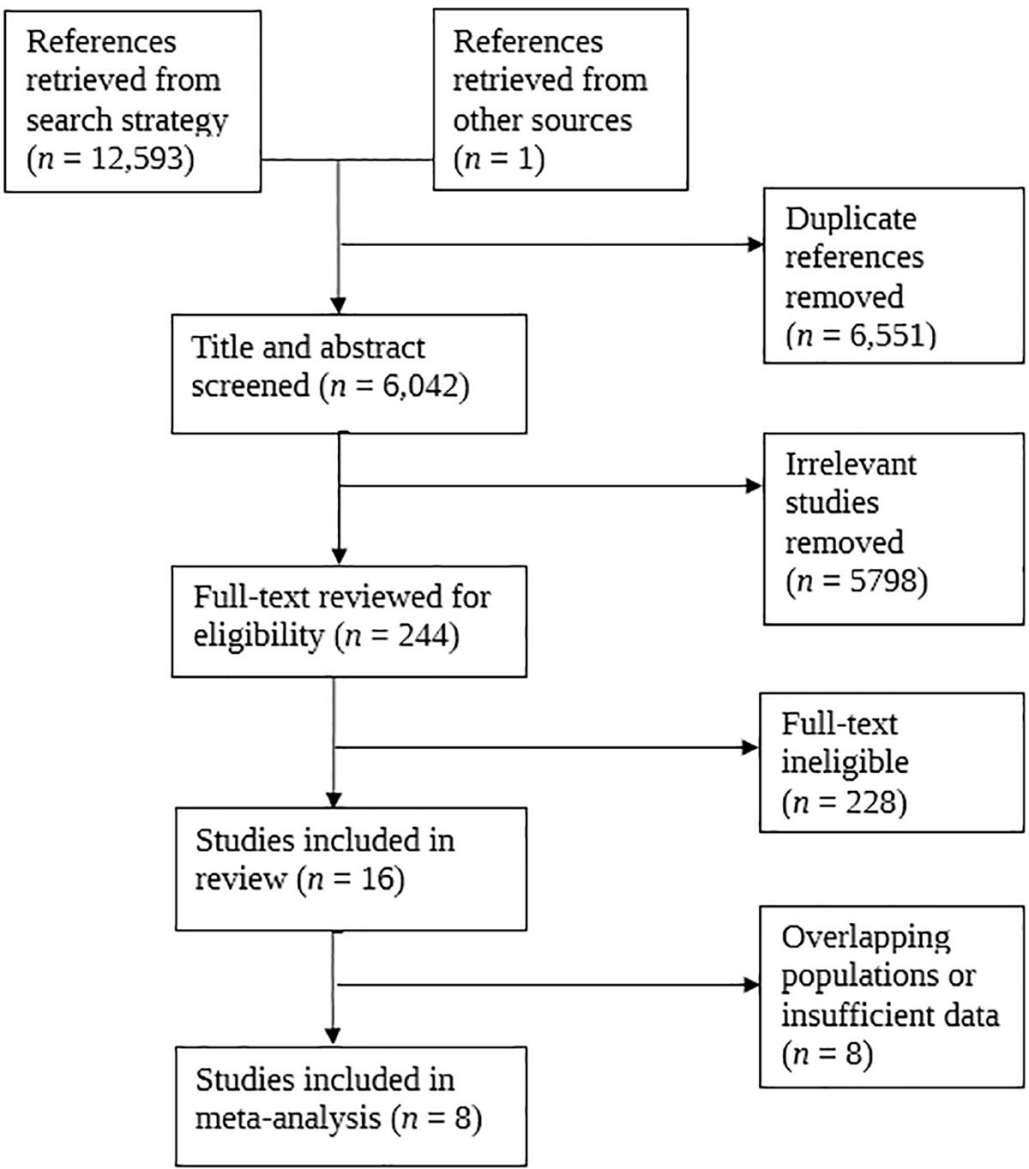
PRISMA flow diagram for articles related to cardiovascular morbidity and mortality among people diagnosed with tuberculosis. PRISMA = Preferred Reporting Items for Systematic Reviews and Meta-Analyses.

We identified 6,042 unique studies through our search strategy, including one identified through personal library search by CAB [35]. After removing irrelevant studies, we assessed the full text of 244 studies for eligibility, excluding 228 studies, keeping 16 studies, and meta-analyzing a subset of estimates extracted from these studies (Fig 1). We included one abstract found in our search as it provided data from a large number of people diagnosed with TB (n = 69,023), was presented at a reputable conference (CHEST), and was not otherwise published [17]. We also included one other study in our review that did not meet all inclusion criteria (did not define CVD [36]) because it involved a population (Russia) not otherwise represented in the review, and merited review in our opinion. However, neither was included in the meta-analyses.

### Characteristics of included studies

Table 1 presents the characteristics of included studies. Fourteen studies examined active TB and CVD morbidity and mortality while two studies investigated latent TB and CVD. The included studies were from a variety of countries, sourcing their analytic samples from TB registries, hospitals, and the general population. Publication dates spanned 2006 to 2018. Eleven countries and one multi-centre cohort are represented in our review, with five studies based on Taiwan’s National Health Insurance Dataset. Four studies sourced individuals from hospitals, seven from the general population, and four from TB registries, while one study did not identify its data source and another sourced data from multiple centres. Follow-up time varied considerably across studies, ranging from 1 to 32 years, but averaged approximately five years (Table 1).

**Table 1:** Characteristics of included studies for systematic literature review of tuberculosis and risk of cardiovascular disease and related mortality.

First Author	Year	Country	Source of TB Data	Enrolment Period	People Diagnosed with TB	Design	Study Objective (page no.)	Follow-up in years: mean / median (max)	ROBINS-I Overall ROB
Bakari	2013	Tanzania	Hospital	2009–2010	34	Case-control	Identify factors (e.g., TB) associated with heart failure in people living with HIV with cardiac complaint (p. 1)	N/A	Serious
Blöndal	2013	Estonia	TB Register	2002–2009	2,449	Cohort	Overall and cause-specific mortality among people with TB and MDR-TB compared to the general population (p. 961)	Mean = 5.3 (9 years)*	Serious
Christensen	2014	Denmark	TB Register	1977–2008	8,291	Cohort	Long-term mortality in TB survivors compared with the general population (p. 406)	Mean = 9.6 (32 years)**	Serious
Chung	2014	Taiwan	Administrative Data	1997–2010	10,168	Cohort	Assess risk of ACS in people with TB compared to controls (p. 80)	Not stated (13 years)	Serious
Giral	2007	France	Hospital	Not Stated	147	Case-control	Analysis of past TB in relation to carotid and femoral atherosclerosis (p. 151)	Mean = 25; SD = 11 between TB diagnosis and sonography	Serious
Hasanain	2018	Egypt	Hospital	2016–2017	54	Case-control	Assess LTBI prevalence among those with/without CAS and evaluate LTBI as predictor of CAS	N/A	Serious
Huaman	2017	United States	Administrative Data	2008–2010	2,026	Cohort	Assess whether TB increased the risk of AMI after adjusting for CVD risk factors (p. 1364)	Not stated (1 year)	Moderate
Huaman	2018	Peru	Hospital	2015–2017	120	Case-control	Assess association between LTBI and AMI (p. 887)	N/A	Serious
Ke	2015	Taiwan	Administrative Data	2008–2010	6,911	Cohort	Analyze CVD and other adverse outcomes after non-chest surgeries in people with pulmonary TB compared to people without TB (p. 2)	Not stated (2 years)	Moderate
Mathew	2006	Russia	TB Register	2002–2003	1,916	Cohort	Risk factors for death during TB treatment (p. 857)	Median = 241 days (censored at death or TB treatment completion)	Serious
Oh	2017	South Korea	Not Stated	2010–2014	69,023	Cohort	Assess incidence of cardiovascular events during TB treatment and if pyrazinamide adds to this risk (p. A199)	Not stated (4 years)	NI
Pettit	2017	International	ART-CC Data Coordinating Centre	1996–2014	2,174	Cohort	Assess the effect of TB (and other ADEs) on non-AIDS mortality risk (p. 2)	Median = 5.18 (IQR: 2.28–9.42 years)	Serious
Sheu	2010	Taiwan	Administrative Data	2000–2003	2,283	Cohort	Assess ischemic stroke risk among people with TB during a 3-year period after diagnosis compared control patients (p. 244)	Not stated (3 years)	Serious
Shuldiner	2016	Israel	TB Register	2000–2010	3,201	Cohort	Assess long-term mortality among TB survivors in Israel, compared to the general population (p. 43)	Median = 5.9 (11 years)	Critical
Wang	2017	Taiwan	Administrative Data	2000–2010	14,350	Cohort	Assess PAD risk in people with TB compared to controls (p. 1671).	Mean = 5.82 (11 years)*	Moderate
Wu	2014	Taiwan	Administrative Data	2001	5,804	Cohort	Assess ischemic stroke after contracting TB (p. 2)	Not stated (3 years)	Serious

ACS = acute coronary syndrome, ADE = AIDS defining event, AIDS = autoimmune deficiency syndrome, AMI = acute myocardial infarction, ART = antiretroviral therapy, ART-CC = Antiretroviral Therapy Cohort Collaboration, CAS = coronary artery stenosis, HIV = human immunodeficiency virus, MDR-TB = multi-drug resistant tuberculosis, NADE = non-AIDS defining event, NI = no information, PAD = peripheral arterial disease, ROBINS-I = Risk of Bias In Non-Randomized Studies of Interventions, ROB = risk of bias, TB = tuberculosis.

*average of means for each group

**average of medians for each group

Table 2 presents all risk estimates extracted from the included studies. Study outcomes included CVD morbidity and mortality, which were primarily defined from ICD coding. One study defined CVD using the third universal definition of myocardial infarction [15], while another defined CVD from multiple, variously coded, centres’ datasets, using the CoDe Project protocol for coding causes of death in HIV [32,37]. People with prevalent (pre-existing) CVD prior to or at time of TB diagnosis were excluded from most studies, with the aim of measuring incident CVD post-TB diagnosis, rather than co-existing TB and CVD. Studies analyzing CVD deaths did not remove people with pre-existing CVD. For the exposure variable, TB, ICD coding was also the most common method of ascertaining exposed persons. However, one study of latent TB infection (LTBI) and acute myocardial infarction (AMI) assessed LTBI using QuantiFERON-TB Gold [15], Bakari et al. used a history of TB in medical records [31], while Oh et al. and Pettit et al. did not provide TB definitions [17,32]. Another LTBI study with coronary artery stenosis (CAS) as the outcome, measured via percutaneous coronary angiography, used both tuberculin skin test (TST>10mm) and QuantiFERON-TB Gold positive as their exposure assessment [38]. People with HIV were variously: included in study populations (two studies included only people living with HIV in their analytic samples [31,32]), excluded from study populations [15,16,38], or included as a proportion of the study population. The models and types of risk estimates varied considerably between studies but all were appropriate to answer our review question. Adjustments were made for age and sex in all studies considered for meta-analysis through either standardization or regression. Additional confounding factors were included in other risk estimates from included studies’ regression analyses.

**Table 2:** Relative risk data extracted from included studies for systematic literature review of tuberculosis and risk of cardiovascular disease and related mortality.

First Author	TB Group (definition)	Prevalent CVD Excluded	HIV Population (percent)	CVD Outcome (definition)	Adjustment Variables	Type	Est^a^	95% CI	
Bakari	All active TB (history of TB)	No	Yes (only HIV included)	Heart failure (echocardiography)	Age, sex, primary education or less, haemoglobin level, CD4 count	OR	3.01	1.32	11.56
Blöndal	Pulmonary-Males (ICD10: A15-A16)	No	Mix (6.1% HIV)	Death from IHD (ICD-10: I20-I25)	Age and sex standardized	SMR	1.80	1.21	2.57
	Pulmonary-Females (ICD10: A15-A16)	No	Mix (3.4% HIV)	Death from IHD (ICD-10: I20-I25)	Age and sex standardized	SMR	3.94	1.28	9.20
	Pulmonary-Males (ICD10: A15-A16)	No	Mix (6.1% HIV)	Death from CBVD (ICD-10: I60-I69)	Age and sex standardized	SMR	2.08	1.04	3.71
	Pulmonary-Females (ICD10: A15-A16)	No	Mix (3.4% HIV)	Death from CBVD (ICD-10: I60-I69)	Age and sex standardized	SMR	3.50	0.72	10.23
Christensen	Pulmonary (ICD8: 011–013; ICD10: A15-A17)	No	Mix (1.7% among TB patients)	Death from CVD (ICD-8: 390–458.99; ICD-10: I00–I99)	Age and sex adjusted	MRR	1.19	1.08	1.31
	Extra-pulmonary (ICD8: 014–019; ICD10: A18-A20)	No	Mix (1.2% among TB patients)	Death from CVD (ICD-8: 390–458.99; ICD-10: I00–I99)	Age and sex adjusted	MRR	1.09	0.92	1.28
Chung	All active TB (ICD9: 011–018)	Yes (excluded anyone with ACS history)	Not stated (presume included)	ACS (ICD9: 410 and 411.1)	Age, sex, hypertension, diabetes, hyperlipidemia, cerebrovascular accident, COPD	HR	1.40	1.14	1.72
Hasanain	LTBI (TST>10mm and QuantiFERON-TB Gold positive on both tests)	No (studied first diagnosis of CAS but did not exclude people with other forms of prevalent CVD)	No (people with HIV excluded)	CAS (percutaneous coronary angiography)	Tobacco smoking, obesity, diabetes, dyslipidemia, metabolic syndrome.	OR	2.50	1.20	17.30
Huaman (2017)	All active TB (ICD9: 010.0–018.9)	Yes (excluded anyone with AMI claim in prior year or same month of TB claim)	No (people with HIV excluded)	AMI (ICD9: 410.0–410.9)	Age, sex, race, diabetes mellitus, hypertension, hyperlipidemia, obesity, tobacco use, CKD, major autoimmune disease	HR	1.98	1.30	3.00
	Pulmonary (not stated)	Yes (excluded anyone with AMI claim in prior year or same month of TB claim)	No (people with HIV excluded)	AMI (ICD9: 410.0–410.9)	Age, sex, race, diabetes mellitus, hypertension, hyperlipidemia, obesity, tobacco use, CKD, major autoimmune disease	HR	2.43	1.50	4.10
Huaman (2018)	LTBI (QuantiFERON-TB Gold)	Yes (only first AMI was studied)	No (people with HIV excluded)	AMI (third universal definition of myocardial infarction)	Age, sex, history of hypertension, history of diabetes mellitus, current tobacco use, history of dyslipidemia, family history of CAD, obesity	OR	1.90	1.05	3.45
Ke	Pulmonary (ICD9: 011)	No	Mix (1.1% HIV of matched cohort)	Stroke (ICD9: 430–438)	Age, sex, low income, urbanization, types of anesthesia, types of surgery, coexisting diseases (anemia, atrial fibrillation, CHF, COPD, diabetes, HIV, IHD, liver cirrhosis, mental disorder, Parkinson’s, PVD, renal dialysis), organ transplantation, steroid use, emergency operation	OR	1.02	0.85	1.21
	Pulmonary (ICD9: 011)	No	Mix (1.1% HIV of matched cohort)	AMI (ICD9: 410)	Age, sex, low income, urbanization, types of anesthesia, types of surgery, coexisting diseases (anemia, atrial fibrillation, CHF, COPD, diabetes, HIV, IHD, liver cirrhosis, mental disorder, Parkinson’s, PVD, renal dialysis), organ transplantation, steroid use, emergency operation	OR	0.89	0.57	1.38
Mathew	All active TB (Tomsk Oblast TB Services)	No	Mix (0.4% HIV)	Vascular disease death (death certificate primary cause of death)	Age and sex standardized	SMR	1.75	1.45	2.09
Oh	All active TB (treated with standard regimen)	Not stated	Not stated	CBVE or AMI (not stated)	Age and sex standardized	SIR	2.89	2.58	3.23
Pettit	All active TB (US-CDC definition of confirmed case)	No	Yes (only HIV included)	Death from CVD (CoDe: 08, 09, 12, 24)	CD4+ count, baseline HIV-1 RNA, sex, HIV transmission risk group, age, year of ART initiation, baseline ART regimen, ADE at or prior to the time of enrollment and ART-CC cohort	HR	2.90	1.57	5.36
Sheu	Non-CNS and non-meningitis TB (ICD9: 010–012, 014–018)	Yes (excluded anyone with stroke prior to index)	Mix (<0.1% HIV)	Ischemic stroke (ICD9: 433–438)	Age, sex, hypertension, diabetes, malignancy, coronary heart disease, hyperlipidemia, monthly income, geographical region, urbanization level, number of CT/MRI scans during follow-up period	HR	1.52	1.21	1.91
	Non-CNS and non-meningitis TB (ICD9: 010–012, 014–018)	Yes (excluded anyone with stroke prior to index)	Mix (<0.1% HIV)	Hemorrhagic stroke (not stated)	Age, sex, hypertension, diabetes, malignancy, coronary heart disease, hyperlipidemia, monthly income, geographical region, urbanization level, number of CT/MRI scans during follow-up period	HR	0.94	0.50	1.79
	Non-CNS and non-meningitis TB (ICD9: 010–012, 014–018)	Yes (excluded anyone with stroke prior to index)	Mix (<0.1% HIV)	Coronary Heart Disease (not stated)	Age, sex, hypertension, diabetes, malignancy, coronary heart disease, hyperlipidemia, monthly income, geographical region, urbanization level, number of CT/MRI scans during follow-up period	HR	1.21	1.08	1.36
Wang	All active TB (ICD9: 010–018)	Yes (excluded anyone with PAD prior to index)	Mix (<1% HIV)	PAD (ICD9: 440.0, 440.2–440.3, 440.8–440.9, 443, 444.0, 444.22, 444.8 and 447.8–447.9)	Age, sex, diabetes mellitus, hypertension, hyperlipidemia, CVD, stroke, COPD, asthma, CKD, HIV, HCV, urbanization level, insured premium	HR	3.93	3.03	4.95
	Pulmonary (ICD9: 011)	Yes (excluded anyone with PAD prior to index)	Mix (<1% HIV)	PAD (ICD9: 440.0, 440.2–440.3, 440.8–440.9, 443, 444.0, 444.22, 444.8 and 447.8–447.9)	Age, sex, diabetes mellitus, hypertension, hyperlipidemia, CVD, stroke, COPD, asthma, CKD, HIV, HCV, urbanization level, insured premium	HR	3.90	2.94	4.86
	Extra-Pulmonary (ICD9: 010, 012–017)	Yes (excluded anyone with PAD prior to index)	Mix (<1% HIV)	PAD (ICD9: 440.0, 440.2–440.3, 440.8–440.9, 443, 444.0, 444.22, 444.8 and 447.8–447.9)	Age, sex, diabetes mellitus, hypertension, hyperlipidemia, CVD, stroke, COPD, asthma, CKD, HIV, HCV, urbanization level, insured premium	HR	2.11	1.31	3.56
	Miliary (ICD9: 018)	Yes (excluded anyone with PAD prior to index)	Mix (<1% HIV)	PAD (ICD9: 440.0, 440.2–440.3, 440.8–440.9, 443, 444.0, 444.22, 444.8 and 447.8–447.9)	Age, sex, diabetes mellitus, hypertension, hyperlipidemia, CVD, stroke, COPD, asthma, CKD, HIV, HCV, urbanization level, insured premium	HR	2.56	0.68	10.49
Wu	Non-CNS and non-meningitis TB (ICD9: 010–012, 014–018)	Yes (excluded anyone with stroke prior to index)	Not stated	Ischemic stroke (ICD9: 433–437)	Age, sex, hypertension, diabetes, atrial fibrillation, chronic rheumatic heart disease, coronary heart disease, other heart disease, hyperlipidemia, monthly income, urbanization level, and geographic region	HR	0.92	0.73	1.14

ACS = acute coronary syndrome, ADE = AIDS defining event, AMI = acute myocardial infarction, ART = anti-retroviral treatment, ART-CC = Antiretroviral Therapy Cohort Collaboration, CAD = coronary artery disease, CAS = coronary artery stenosis, CBVD = cerebrovascular disease, CBVE = cerebrovascular event, CHF = congestive heart failure, CI = confidence interval, CKD = chronic kidney disease, CNS = central nervous system, CoDe = Coding of Death in HIV Project, COPD = chronic obstructive pulmonary disorder, CVD = cardiovascular disease, CT = computed tomography, Est. = estimate, HCV = hepatitis C virus, HIV = human immunodeficiency virus, HR = hazard ratio, IHD = ischemic heart disease, LTBI = latent tuberculosis infection, MRI = magnetic resonance imaging, MRR = mortality rate ratio, NI = no information, OR = odds ratio, PAD = peripheral arterial disease, PVD = peripheral vascular disease, TB = tuberculosis, SIR = standardized incidence ratio, SMR = standardized mortality ratio.

^a^All estimates (Est.) are adjusted for (or standardized by) variables listed in “Adjustment Variables” column.

### Data synthesis and analysis

A subset of included studies (*n* = 8) were meta-analyzed [15,16,31,32,38–41]. Because of overlapping populations from studies in Taiwan, we included only the study with the largest sample [41], removing others [14,33,35,42]. Blöndal et al. reported two relevant outcomes, death from ischaemic heart disease and death from cerebrovascular event, with sex-specific estimates for each [39]; therefore, we included one outcome, ischemic heart disease death, including both sex-specific estimates, in our per-protocol meta-analysis. Wang et al. and Huaman et al. (2017) reported TB type-specific estimates but we opted to use the overall TB estimates as they were more stable and in line with our protocol [41,43]. We excluded from all meta-analyses two studies (Giral et al., and Shuldiner et al.) that presented only frequency tables for CVD outcomes [44,45]. We excluded Mathew et al. from the meta-analyses as the authors did not present a definition of CVD and their study contained only 4 deaths from CVD among TB patients [36], excluded Oh et al. as they did not provide definitions for TB or CVD [17], and excluded the estimate for females from Blondal et al., as its confidence interval was asymmetrical [39].

**Fig 2:**
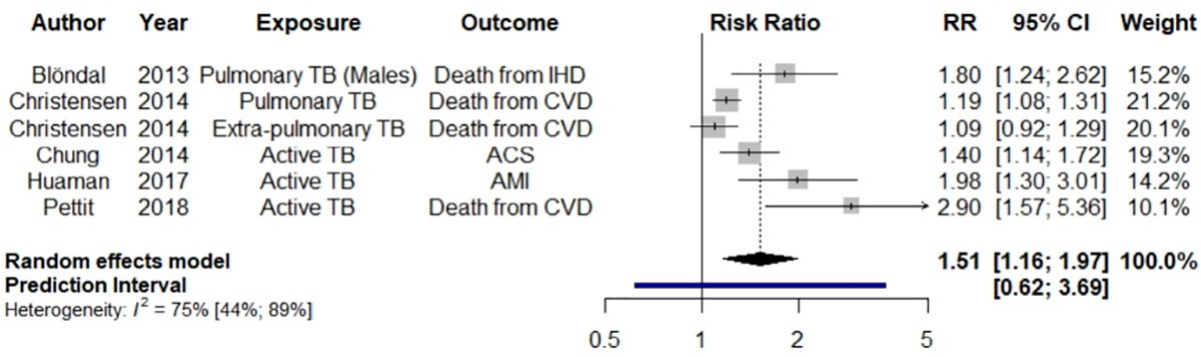
Forest plot of *post hoc* random effects meta-analysis results for systematic literature review of tuberculosis and risk of cardiovascular disease and related mortality. ACS = acute coronary syndrome, AMI = acute myocardial infarction, CBVE = cerebrovascular event, CI = confidence interval, CVD = cardiovascular disease, IHD = ischemic heart disease, RR = risk ratio, TB = tuberculosis. Black diamond = pooled RR and 95% confidence interval; blue bar = 95% prediction interval.

Fig 2 presents our *post hoc* meta-analysis results for MACE among persons diagnosed with active TB (pooled RR = 1.51; 95% CI 1.16–1.97, p = 0.0024), with *I*^2^ = 75.3%, and prediction interval of 0.62 to 3.69. For the *post hoc* meta-analysis, we removed the studies of latent TB [14,37]. We then pooled studies reporting MACE [31,41]. For this analysis, in place of Wang et al. (outcome: peripheral arterial disease) [41], we substituted the second largest study from Taiwan, which analyzed AMI and unstable angina as the combined endpoint of acute coronary syndrome [46]. We consider this meta-analysis the main analysis as it is more interpretable due to more harmonized definitions of TB and CVD.

**Fig 3:**
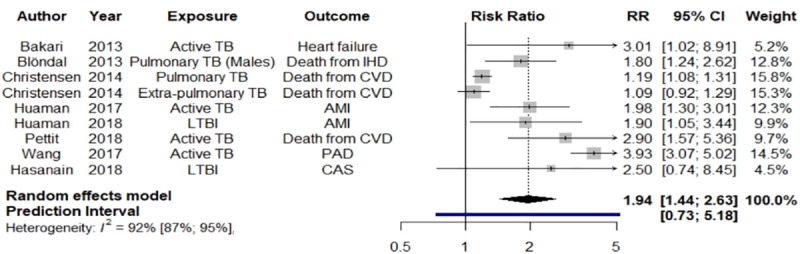
Forest plot of per-protocol meta-analysis for systematic literature review of tuberculosis and risk of cardiovascular disease and related mortality. AMI = acute myocardial infarction, CAS = coronary artery stenosis, CBVE = cerebrovascular event, CI = confidence interval, CVD = cardiovascular disease, IHD = ischemic heart disease, PAD = peripheral arterial disease, RR = risk ratio, TB = tuberculosis. Black diamond = pooled RR and 95% confidence interval; blue bar = 95% prediction interval.

Fig 3 presents our per-protocol meta-analysis results of CVD morbidity and mortality risk among persons diagnosed with TB. The pooled RR of CVD morbidity and mortality among persons diagnosed with TB was 1.94 (95% CI 1.44–2.67, p<0.0001). Significant heterogeneity was found between estimates (*I*^2^ = 92%), and this heterogeneity is reflected in the prediction interval of 0.73 to 5.18.

### Risk of bias within and across studies

**Fig 4:**
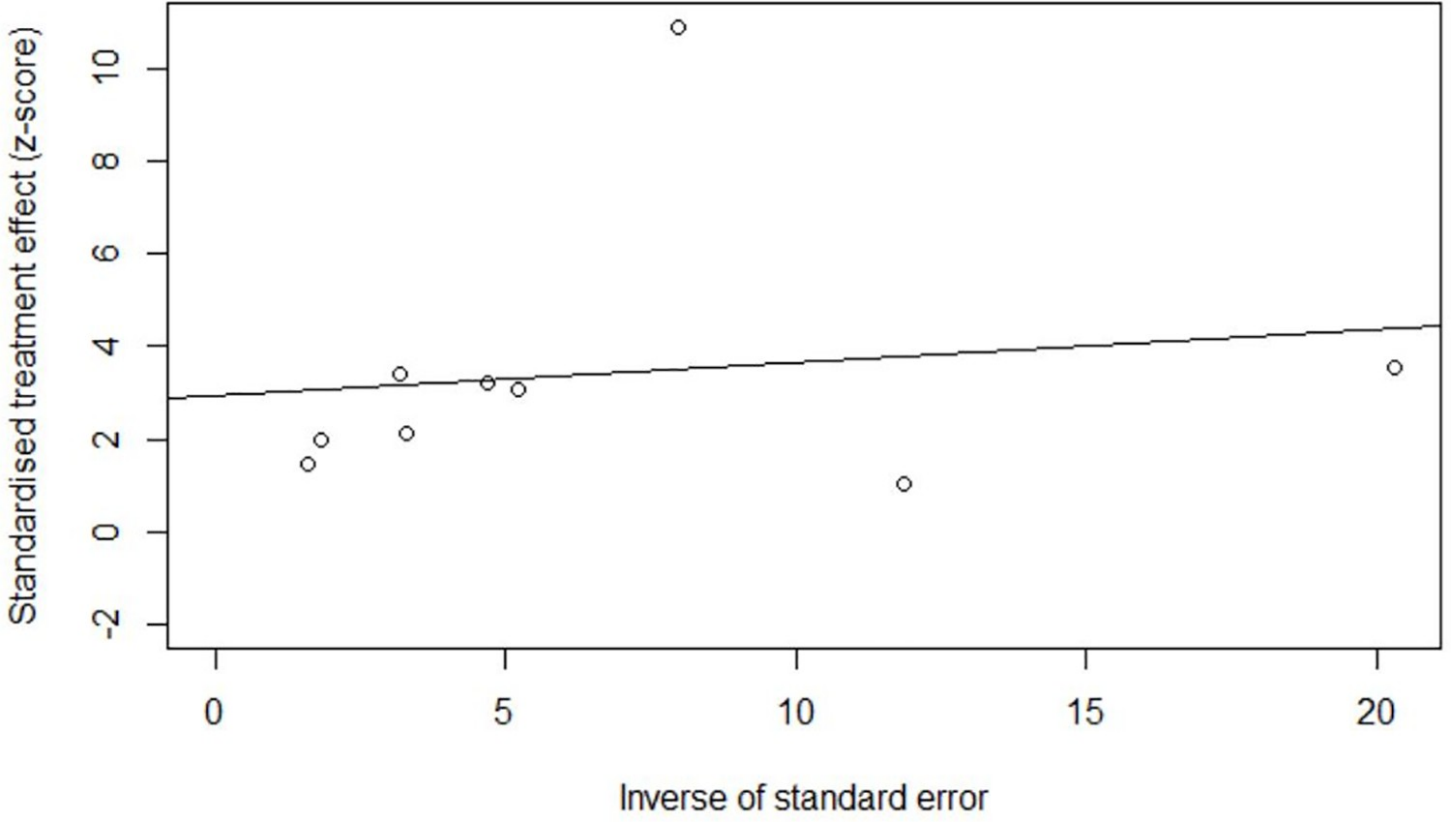
Publication bias assessment: Radial plot for per-protocol meta-analysis of tuberculosis and risk of cardiovascular disease and related mortality.

**Fig 5:**
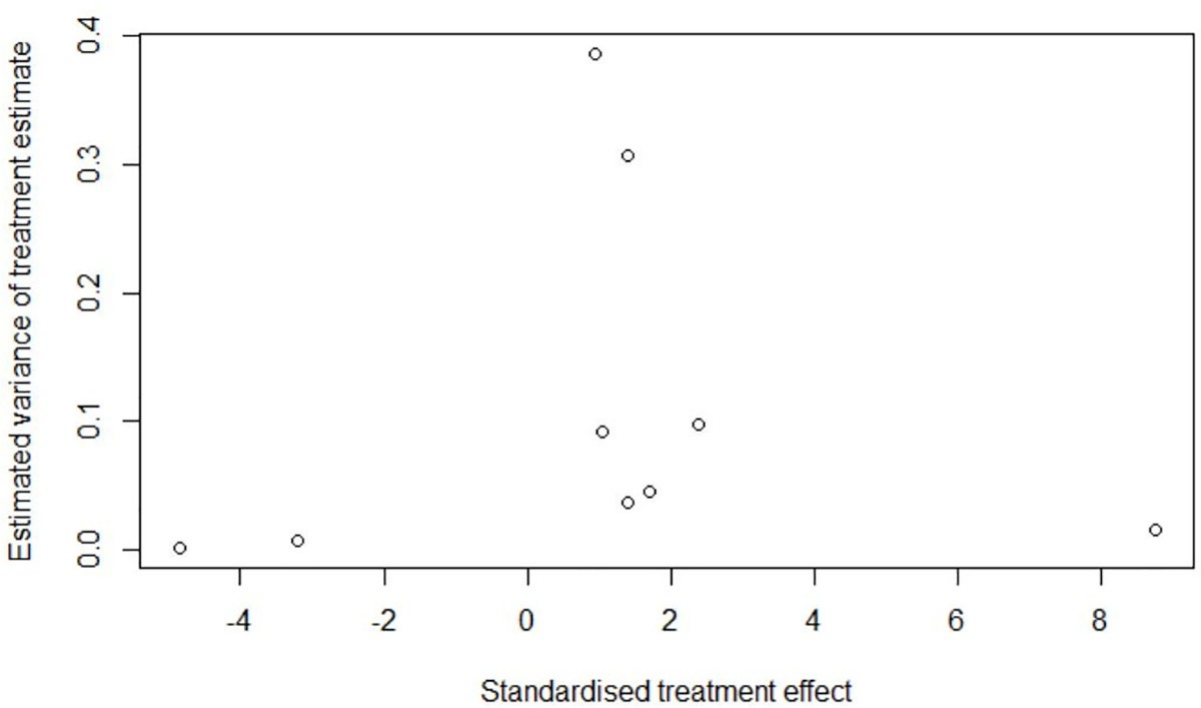
Publication bias assessment: Funnel plot for per-protocol meta-analysis of tuberculosis and risk of cardiovascular disease and related mortality.

One study had insufficient information to judge RoB, while the remaining were judged at ‘critical’ (n = 1), ‘serious’ (n = 11), ‘moderate’ (n = 3), or ‘no information’ (n = 1) for RoB using the ROBINS-I tool (Table 1). The mode of the RoB of individual studies in the confounding domain was ‘serious’, which determined the overall RoB of most studies as ‘serious’, based on the ROBINS-I guidance [26]. We estimated the pooled RoB across studies to be ‘serious’. Radial and funnel plots for the per-protocol analysis indicated potential publication bias (Figs 4 and 5), yet insufficient evidence to reject the null hypothesis of symmetry (linear regression test: *p* = 0.1095; rank correlation test: *p* = 0.5316).

**Fig 6:**
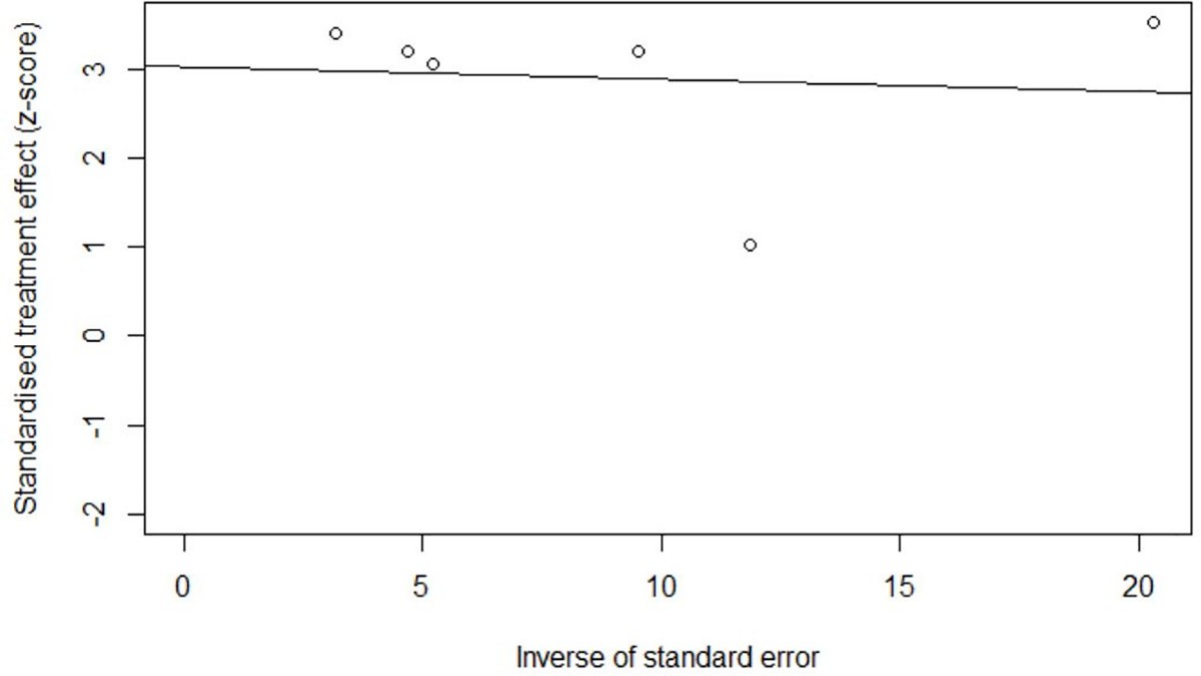
Publication bias assessment: Radial plot for *post hoc* meta-analysis of tuberculosis and risk of cardiovascular disease and related mortality.

**Fig 7:**
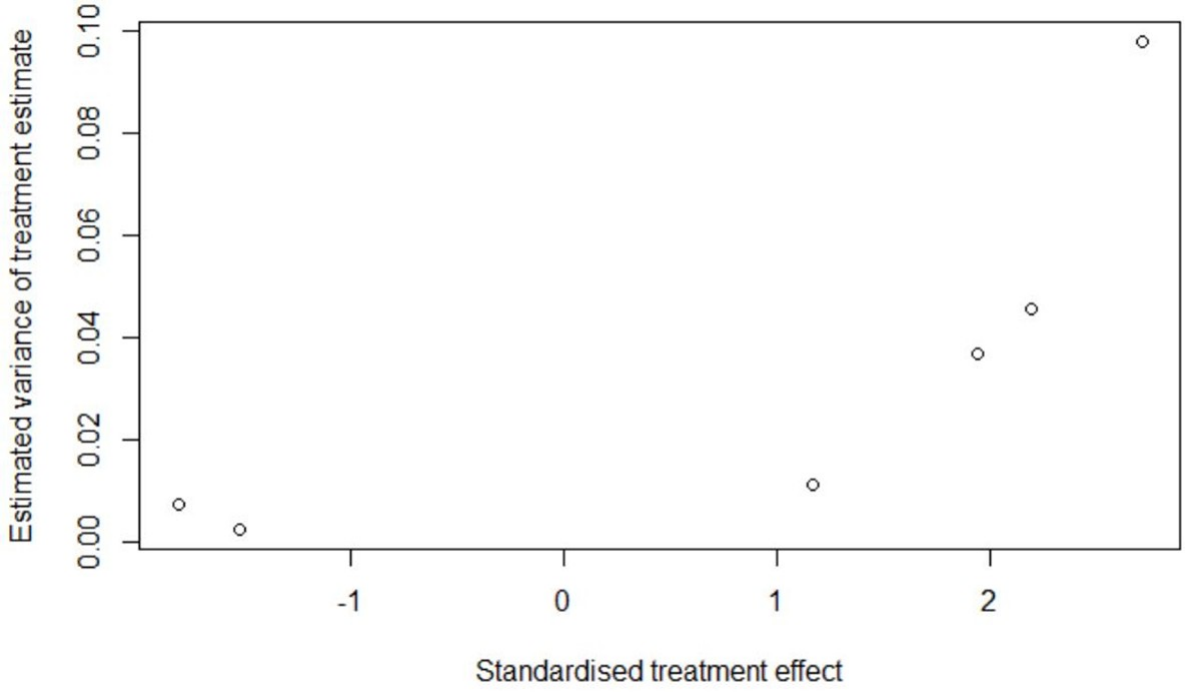
Publication bias assessment: Funnel plot for *post hoc* meta-analysis of tuberculosis and risk of cardiovascular disease and related mortality.

However, when the post hoc meta-analysis was subjected to these tests, we observed asymmetry (Fig 6 and Fig 7) with sufficient evidence to conclude that the hypothesis of no publication bias could be rejected (linear regression test: *p* = 0.0186; rank correlation test: *p* = 0.0146)

### Sensitivity and subgroup analyses

The finding of elevated CVD morbidity and mortality from both the per-protocol and post hoc meta-analyses were robust to inclusion/exclusion of: estimates from studies enrolling populations requiring a medical condition beyond TB (people living with HIV [31,32], hospitalized persons [15], and non-chest surgery patients [33]); estimates that were not adjusted for risk factors beyond age and sex [15,16,31,32,41]; and the estimate from a study of extrapulmonary TB and CVD mortality [40] with asymmetrical confidence intervals (Table 3). Sub-group analyses showed lower pooled RRs from the per-protocol meta-analysis, for both CVD mortality [32,36,39,40], and CVD events [15,16,35], yet were consistent with the *post hoc* meta-analysis pooled RR, although the CVD events sub-group analysis was not statistically significant (p = 0.1557). Prediction intervals for all meta-analyses showed wide heterogeneity in potential RRs for patients with TB and future studies of TB and risk of CVD morbidity and mortality.

**Table 3:** Sensitivity and sub-group random effects meta-analyses of tuberculosis and risk of cardiovascular disease and related mortality.

Sensitivity / Sub-Group Meta-Analysis	Pooled RR	95% CI	95% PI	p-value
Removed studies requiring a medical condition other than TB for inclusion from per-protocol meta-analysis	1.77	1.12–2.7800	0.31–10.17	0.0140
Removed non-RF-adjusted estimates from per-protocol meta-analysis	2.78	2.12–3.63	1.46–5.29	<0.0001
Removed extra-pulmonary TB estimate from per-protocol meta-analysis.	2.22	1.66–2.95	0.93–5.24	<0.001
Sub-group: cardiovascular death only	1.70	1.09–2.67	.034–8.66	0.0205
Sub-group: cardiovascular events only	1.44	0.87–2.40	0.0036–584.38	0.1557

CI = confidence interval, PI = prediction interval, RF = risk factor, RR = risk ratio, TB = tuberculosis.

## Discussion

To our knowledge, this is the first systematic review to examine the relationship between TB and risk of CVD morbidity and mortality. Our findings indicate that persons diagnosed with TB are at elevated risk of CVD morbidity and mortality compared to persons not diagnosed with TB. Sensitivity and sub-group analyses had consistent conclusions. The significant and positive association observed between TB and CVD risk may be causal or proximal in nature due to the limitations of the studies included, as noted below. A pooled 51% increased for MACE among people diagnosed with TB compared to non-TB controls was found (95% CI: 16–97%). Because only two reviewed study examined LTBI and CVD [38,43] our findings are not generalizable beyond active TB.

Our finding of increased CVD risk among people diagnosed with TB is consistent with the literature. A related review of the relationship between acute infections and AMI showed pooled RRs for CVD events in persons diagnosed with pneumonia compared to those without pneumonia ranged from 3.2 to 6.3 during the first four weeks from infection onset, with long-term (1 to 4 years post-infection onset) RRs ranging from 1.5 to 2.5 [11]. A systematic review of TB and all-cause mortality found a similarly elevated mortality rate among those diagnosed with TB (pooled standardized mortality ratio = 2.91; 95% CI 2.21–3.84), for which the authors attributed 20% (95% CI 15–26) to CVD [47]. These findings are consistent with our meta-analytic conclusion of increased risk, but our pooled effect size was considerably lower than that for post-TB mortality.

### Strengths and limitations

From an epidemiological point of view, there are important strengths yet critical limitations to our review. The search strategy was limited to English language studies. The pooled RR estimate included a wide range of CVD outcomes from diverse source populations, including a mix of pulmonary and extra-pulmonary forms of TB, examined through a variety of designs and statistical models producing a variety of effect estimates. While this review contains many types of CVD morbidity and mortality, we view this as a strength: guarding against underestimation of TB’s role as a marker for CVD risk. Most reviewed studies adjusted for multiple potential confounders.

Within included studies, lack of control for smoking was a key source of potential bias and thus affected our review-level pooled RoB estimate. However, three studies adjusted for current tobacco use all of which found increased CVD risk among those diagnosed with TB [15,16,38]. The adjustment for chronic obstructive pulmonary disease in two studies provided an indirect form of adjustment for confounding by smoking [41,46]. The effects of other infectious diseases (e.g., HIV) and socioeconomic status were also potential sources of bias that were not adjusted for in all analyses. Income and socioeconomic status generally are related to CVD risk. Adjustment for income was made in three of the reviewed studies [14,35,41]. Two of the studies, Sheu et al. and Wang et al. [14,41], found a significant association between TB and CVD, and one of the studies, Wu et al., had a null finding [35]. Bakari et al. adjusted for education in their analysis of heart failure among patients with a history of TB compared to no TB history, and found a significant association between TB and CVD[31].

These limitations preclude unbiased causal inference about the association between TB and CVD. However, they were considered in the ROBINS-I assessments and our conclusion about the significant positive association between TB and incident CVD can be tempered by the ‘Serious’ RoB assigned to our pooled RR estimate. Moreover, concern about lack of adjustment for these factors, or residual confounding, is less important when viewing our pooled RR as evidence of TB being a marker for CVD risk. However, critical knowledge gaps and study design challenges remain for future studies seeking to make causal inferences about TB and CVD risk, especially given that study data will largely remain observational in nature [48,49].

### Biological and clinical considerations

The mounting evidence for increased noncommunicable disease risk among TB patients has prompted numerous hypotheses and studies of potential mechanisms for the multi-directional relationships between TB and noncommunicable diseases [48,49]. There are a number of potential biological mechanisms relating TB to CVD, although none are definitive. It is possible that there are common biological mechanisms between TB and pneumonia in generating excess CVD risk [10–12]. In their narrative review, Huaman et al. summarized hypothesized biological mechanisms of CVD in people infected by TB as follows: direct effects on the myocardium or coronary arteries; pro-inflammatory cytokine expression; immune activation via macrophages and monocytes or CD4^+^, TH1 and TH7 cells; and auto-immune mediation through mycobacterial heat-shock protein 65 antibodies [12]. These mechanisms are consistent with literature on CVD in infectious diseases such as pneumonia, hepatitis C, and HIV [10,15,38,45]. These mechanisms are proposed to contribute to atherosclerotic plaque development over the long term, and to short-term increases in risk of cardiovascular events such as AMI [10–12,46]. In contrast, Giral et al. found no evidence of increased atherosclerosis among patients with a history of TB compared with those without a history of TB, although with a limited sample [45].

Review-level conclusions about the epidemiological association between LTBI and CVD cannot be reached from the two included studies. However, TB exists on a continuum between LTBI and active TB, with an unknown latency period that could last a few months to a few decades [50]. LTBI involves continual production and clearance of *M*. *Tuberculosis* within the host and involves multiple potential sites in the body, with various host-organism immune interactions. These mechanisms are acting throughout the latent period, which may lead to long-term cardiovascular damage that manifests in adverse cardiovascular outcomes after active TB disease develops, similar, to some degree, as the hypothesized role of LTBI in development of diabetes mellitus [48].

Although the evidence for CVD risk among persons diagnosed with TB is seriously limited from a causal inference perspective, we believe it is appropriate to adopt a precautionary approach to this evidence. A precautionary approach to CVD in TB care means that programs and practitioners consider the risks of not acting to prevent CVD morbidity and mortality until more complete evidence of increased risk is produced, versus the potential practical benefits for TB patients of implementing CVD prevention strategies based on provisional evidence, reasonable suspicion of risk, and often irreversible harm [51]. A first step may be to ensure existing CVD is diagnosed and known CVD risk factors prevalent among persons diagnosed with TB (for example, exposure to tobacco smoke [52], alcohol consumption [53], and diabetes [54]) are identified and managed.

CVD risk assessment among persons diagnosed with TB should follow current guidelines and evidence regarding screening asymptomatic adults [55]. There is likely limited risk to TB patients in screening for CVD and modifiable CVD risk factors, although randomized trial risk-benefit data for systematic CVD screening among TB patients is currently unavailable. In HIV medicine, CVD screening among people living with HIV has been incorporated into treatment guidelines [19,20]. A scientific statement was recently issued by the American Heart Association reviewing the mounting evidence for the risk of CVD, as well as prevention and management strategies, for people living with HIV [56]. In this statement, the REPRIEVE trial for vascular event prevention among people living with HIV is mentioned for its potential to provide the first RCT evidence of statins’ efficacy in CVD prevention among people living with HIV [57].

In TB medicine, statins have potential for improving TB treatment outcomes [58], and are being considered as host-directed therapy in two trials where they are used as adjuvants to TB chemotherapy [59–61]. While not designed to assess the efficacy of statins for CVD prevention in TB patients, dose-safety data from these trials may inform future trials for TB patients with such a goal, similar, perhaps, to the REPRIEVE trial for HIV patients [57]. In the absence of RCT evidence of statin efficacy for CVD prevention in TB, a subgroup analysis of people with a history of TB within the REPRIEVE trial might provide estimates of statins’ effectiveness for reducing CVD risk in people living with HIV with a history of TB. Retrospective pharmacoepidemiological studies of people diagnosed with TB who have received statin therapy may also provide observational evidence regarding statins’ potential for reducing CVD risk in TB.

## Conclusions

This paper reviewed the epidemiological evidence for increased risk of incident CVD among TB patients. Our meta-analysis suggests that a diagnosis of TB is a marker for elevated risk of CVD. This finding has implications for TB research and care: physicians treating patients with a diagnosis of active TB may consider these patients at elevated risk of CVD; hypothesized mechanisms leading to increased risk of CVD among persons diagnosed with TB could be examined prospectively; TB programs and care providers may also consider offering cardiovascular health assessment to persons diagnosed with active TB, guided by current CVD screening guidelines. While further research is needed that addresses the limitations of existing studies, by considering these implications, TB programs and care providers may be able to improve cardiovascular outcomes for people affected by TB.

## Supporting information

S1 Table EMBASE database search for systematic review of tuberculosis and the risk of cardiovascular disease and related mortality. (DOCX)

S2 Table MEDLINE database search for systematic review of tuberculosis and the risk of cardiovascular disease and related mortality. (DOCX)

S3 Table MEDLINE database search for systematic review of tuberculosis and the risk of cardiovascular disease and related mortality. (DOCX)

S1 Appendix (DOCX)
