# Supplementary material for: Cardiovascular morbidity and mortality among persons diagnosed with tuberculosis: A systematic review and meta-analysis
Source: PLoS One. 2020 Jul 10;15(7):e0235821. doi: 10.1371/journal.pone.0235821 (PMC7351210; doi:10.1371/journal.pone.0235821)
Supplement: S1 Table — (DOCX) [file pone.0235821.s001.docx]

**S1** **Table. EMBASE database search for systematic review of tuberculosis and the risk of cardiovascular disease and related mortality**

| **Number** | **Search** | **Records** |
| --- | --- | --- |
| 1 | exp tuberculosis/ | 184989 |
| 2 | (tuberculosis or mycobacterium tuberculosis or TB or mycobacterial infection or TBC or MDR-TB or LTBI).tw. | 200178 |
| 3 | 1 or 2 | 249919 |
| 4 | exp cardiovascular disease/ or cardiovascular dis*.tw. | 3978199 |
| 5 | (coronary or myocard* or ischem* or ischaem* or stroke or cerebrovasc* or cerebral vascular or peripheral arter* or angina).tw. | 1497943 |
| 6 | 4 or 5 | 4257165 |
| 7 | 3 and 6 | 22485 |
| 8 | randomized controlled trial/ | 587336 |
| 9 | clinical study/ or exp case control study/ or exp clinical trial/ or exp longitudinal study/ or exp prospective study/ or exp retrospective study/ | 3051255 |
| 10 | (random* or cohort or case control or RCT).tw. | 2414305 |
| 11 | 8 or 9 or 10 | 4424075 |
| 12 | 7 and 11 | 5433 |
| 13 | 12 not ((exp animal/ or nonhuman/) not exp human/) | 5396 |
| 14 | vasc*.ti,ab. | 902815 |
| 15 | 4 or 5 or 14 | 4664088 |
| 16 | 3 and 15 | 23891 |
| 17 | 11 and 16 | 5645 |
| 18 | peripheral vascular disease/ or exp vascular disease/ | 2377903 |
| 19 | 1 and 3 | 184989 |
| 20 | 3 and 18 | 12151 |
| 21 | 6 or 14 or 18 | 4664088 |
| 22 | 3 and 21 | 23891 |
| 23 | 11 and 22 | 5645 |
| 24 | 13 and 23 | 5396 |

**Notes:** EMBASE 1974 to 2020 January 10 (search date January 19, 2020).
