# Supplementary material for: Cardiovascular morbidity and mortality among persons diagnosed with tuberculosis: A systematic review and meta-analysis
Source: PLoS One. 2020 Jul 10;15(7):e0235821. doi: 10.1371/journal.pone.0235821 (PMC7351210; doi:10.1371/journal.pone.0235821)
Supplement: S2 Table — (DOCX) [file pone.0235821.s002.docx]

**S2 Table. MEDLINE database search for systematic review of tuberculosis and the risk of cardiovascular disease and related mortality**

| **Number** | **Search** | **Records** |
| --- | --- | --- |
| 1 | exp tuberculosis/ | 192086 |
| 2 | (tuberculosis or mycobacterium tuberculosis or TB or mycobacterial infection or TBC or MDR-TB or LTBI).tw. | 204779 |
| 3 | 1 or 2 | 263653 |
| 4 | exp cardiovascular disease/ or cardiovascular dis*.tw. | 2404799 |
| 5 | (coronary or myocard* or ischem* or ischaem* or stroke or cerebrovasc* or cerebral vascular or peripheral arter* or angina).tw. | 1064046 |
| 6 | 4 or 5 | 2733612 |
| 7 | 3 and 6 | 8827 |
| 8 | randomized controlled trial/ | 498940 |
| 9 | clinical study/ or exp case control study/ or exp clinical trial/ or exp longitudinal study/ or exp prospective study/ or exp retrospective study/ | 2328426 |
| 10 | (random* or cohort or case control or RCT).tw. | 1659803 |
| 11 | 8 or 9 or 10 | 3270287 |
| 12 | 7 and 11 | 1055 |
| 13 | 12 not ((exp animal/ or nonhuman/) not exp human/) | 1042 |
| 14 | vasc*.ti,ab. | 658166 |
| 15 | 4 or 5 or 14 | 3123047 |
| 16 | 3 and 15 | 10050 |
| 17 | 11 and 16 | 1259 |
| 18 | peripheral vascular disease/ or exp vascular disease/ | 1634216 |
| 19 | 6 or 14 or 18 | 3123047 |
| 20 | 3 and 19 | 10050 |
| 21 | 11 and 20 | 1259 |
| 22 | 13 and 21 | 1042 |

**Notes:** Ovid MEDLINE(R) Epub Ahead of Print, In-Process & Other Non-Indexed Citations, Ovid MEDLINE(R) Daily and Ovid MEDLINE(R) 1946 to January 17 (search date: January 19, 2020).
